# Brain activity during pursuit and goal-conflict threat avoidance in major depressive disorder

**DOI:** 10.1017/pen.2022.1

**Published:** 2022-08-23

**Authors:** Lindsey Marwood, Toby Wise, Jess Kerr-Gaffney, Rebecca Strawbridge, Steve C R Williams, Anthony J Cleare, Adam Perkins

**Affiliations:** 1 Centre for Affective Disorders, Department of Psychological Medicine, Institute of Psychiatry, Psychology & Neuroscience, King’s College London, DE Crespigny Park, London, UK; 2 Department of Neuroimaging, Institute of Psychiatry, Psychology and Neuroscience, King’s College London, London, UK; 3 South London and Maudsley NHS Foundation Trust, London, UK

**Keywords:** Depression, fMRI, Threat, Fear, Anxiety, Goal-conflict

## Abstract

Threat avoidance is a prominent symptom of affective disorders, yet its biological basis remains poorly understood. Here, we used a validated task, the Joystick Operated Runway Task (JORT), combined with fMRI, to explore whether abnormal function in neural circuits responsible for avoidance underlies these symptoms. Eighteen individuals with major depressive disorder (MDD) and 17 unaffected controls underwent the task, which involved using physical effort to avoid threatening stimuli, paired with mild electric shocks on certain trials. Activity during anticipation and avoidance of threats was explored and compared between groups. Anticipation of aversive stimuli was associated with significant activation in the dorsal anterior cingulate cortex, superior frontal gyrus, and striatum, while active avoidance of aversive stimuli was associated with activity in dorsal anterior cingulate cortex, insula, and prefrontal cortex. There were no significant group differences in neural activity or behavioral performance on the JORT; however, participants with depression reported more dread while being chased on the task. The JORT effectively identified neural systems involved in avoidance and anticipation of aversive stimuli. However, the absence of significant differences in behavioral performance and activation between depressed and non-depressed groups suggests that MDD is not associated with abnormal function in these networks. Future research should investigate the basis of passive avoidance in major depression. Further, the JORT should be explored in patients with anxiety disorders, where threat avoidance may be a more prominent characteristic of the disorder.

Major depressive disorder (MDD) is highly prevalent and greatly impacts quality of life, affecting mood, physical health, social functioning, and cognition (Cambridge, Knight, Mills, & Baune, 2018; Wells et al., 1989; Wittchen et al., 2011). Currently, psychiatric assessments are based on patient self-report and observation of patient behaviors without parallel measurement of underlying mechanisms. The development of robust neuropsychological measures – linking key dimensions of mental health to their underlying neural circuitry – could be a key step in achieving a more evidence-based approach to psychiatric treatment.

Threat avoidance is one such measurable domain of functioning. It is an innate defensive reaction to potential threats and an evolved survival behavior (Mobbs, Hagan, Dalgleish, Silston, & Prevost, 2015). Problems may arise when these avoidance behaviors lose their adaptive qualities, preventing people from learning which situations are and are not dangerous, becoming habitual or excessive (LeDoux, Moscarello, Sears, & Campese, 2017). Trew (2011) proposed that avoidance and decreased approach to threat contribute to the maintenance of depression through the development of negative information processing biases and reduction in exposure to positive situations.

Active threat avoidance behaviors (i.e., threats requiring action for their avoidance) can be differentiated in terms of their defensive direction. Fear is associated with orientation *away* from pursuing threats (where escape is possible, leading to flight behavior), whereas anxiety is associated with more ambiguous threats which require investigation and necessitate orientation *towards* threat (Gray & McNaughton, 2000). Defensive direction was traditionally studied in isolation in humans until the development of the Joystick Operated Runway Task (JORT), which allows within-task, within-subject comparison of fear and anxiety (Perkins et al., 2009). The JORT is an adaptation of the Mouse Defence Test Battery (Blanchard, Griebel, & Blanchard, 2003), an established active-avoidance model in rodents. In the human translation, participants are chased by digital predators under two conditions: pursuit trials when the participant’s cursor is chased by one predator requiring flight behavior and goal-conflict conditions which contain two predators moving forward (one in front of the cursor and one behind) leading to approach-avoidance conflict as the participant must move fast enough to avoid the latter but not so fast as to collide with the former. On 50% of trials (threat condition), electric shocks are administered if the cursor is caught by a predator, adding an additional element of threat by association with the cued aversive event.

An fMRI version of the JORT has been piloted in healthy participants to facilitate understanding of the brain systems underlying fear and anxiety (Perkins et al., 2019). In line with previous research (Bach et al., 2014; Mobbs et al., 2007, 2009), differentiations indicated that midbrain and prefrontal activation are associated with pursuit and hippocampal activation associated with goal conflict. Perkins et al. (2019) also found that lower hippocampal activation during goal-conflict plus threat was associated with higher neuroticism scores, suggesting that those with a personality more susceptible to affective disorders have altered goal-conflict processing under threat. Brain activity associated with both goal conflict and pursuit during threat avoidance has yet to be studied in clinical populations.

Thus, the current study aimed to administer the JORT in patients with MDD to examine the neural correlates of threat avoidance in this population compared to healthy controls. Due to findings linking affective disorders with elevated attentional focus on threats and negative anticipation (Grupe & Nitschke, 2013), brain activation during anticipation (when the type of task was cued) was evaluated, a previously unstudied phase of the task. It was hypothesized that anticipation would be associated with increased activity in the anterior cingulate cortex (ACC), ventromedial prefrontal cortex (vmPFC), and striatum (Critchley, Mathias, & Dolan, 2001; Mobbs et al., 2007; Rzepa, Fisk, & McCabe, 2017), regions related to cognitive processing of emotions such as fear, evaluation of context, vigilance, and behavioral control (Amat, Paul, Zarza, Watkins, & Maier, 2006; Critchley, Wiens, Rotshtein, Öhman, & Dolan, 2004; Liotti et al., 2000; Mobbs et al., 2009; Schiller, Levy, Niv, LeDoux, & Phelps, 2008).

It was hypothesized that active avoidance in pursuit trials (relating to fear) would elicit activity in the vmPFC, cerebellum, and periaqueductal gray (PAG) (Mobbs et al., 2007, 2009; Perkins et al., 2019) and that active avoidance in goal-conflict (anxiety-related) trials would elicit hippocampal activation (Bach et al., 2014), while those with MDD would show increased activation compared to controls in the regions hypothesized for both anticipation and threat avoidance conditions. An equivalent prediction was made for the main effects of each condition and association with threat (comparison between threat versus no threat trials). As well as whole brain analyses, region of interest (ROI) analyses were conducted with the expectation that: 1) PAG activity would be positively associated with threat proximity and 2) goal-conflict sensitivity would be associated with activation of the anterior hippocampus and that this activation would be positively associated with neuroticism, according to findings in healthy controls (Perkins et al., 2019).

Thirdly, relationships between behavioral JORT performance, neural activation, and psychological factors were explored. JORT behavioral measures included: Flight Intensity (FI) (the degree to which signaled threat increased speed of movement during pursuit) and Risk Assessment Intensity (RAI) (the degree to which signaled threat increased anxiety behavior, i.e., oscillations in movement during goal-conflict). It was hypothesized that trait anxiety would relate to neural activity on goal-conflict trials and self-reported fear would correlate with neural activity on pursuit trials (Perkins et al., 2013; Perkins, Kemp, & Corr, 2007; Perkins & Corr, 2006). Subjective dread ratings were anticipated to positively correlate with the anticipation phase and RAI on goal-conflict trials (Berns et al., 2006). Elevated JORT threat-avoidance behaviors (FI and RAI) were hypothesized to be associated with exaggerated activation patterns in the hypothesized regions.

## 1. Methods

### 1.1 Participants

Thirty-nine right-handed participants (20 MDD, 19 controls) aged 18–65 years were recruited from waiting lists of South London psychological therapy services and online advertisements (Wise et al., 2016). Participants were required to meet current DSM-IV criteria (American Psychiatric Association, 2000) for MDD, as determined by the Mini International Neuropsychiatric Interview (MINI) (Sheehan et al., 1998). A score of ≥14 on the 17-item Hamilton Depression Rating Scale (Hamilton, 1960) was required for inclusion. A diagnosis of bipolar disorder or current psychosis (assessed using the MINI), or borderline personality disorder (determined via the Structured Clinical Interview for DSM-IV Axis II disorders; First et al., 1997) were exclusionary. Comorbid Axis I anxiety disorders were allowed.

Participants were not receiving any form of treatment (psychotropic or psychological) at the time of scanning and had been psychotropic medication-free for at least 8 weeks prior to inclusion. Age, gender, and handedness-matched controls were assessed to exclude personal and familial (first-degree relative) psychiatric history.

Exclusion criteria for all participants included: neurological disorders, learning disabilities, uncorrectable visual problems, illicit substance use in the preceding two months, current (within 12 months) alcohol or other substance use disorder (MINI), unstable or severe medical conditions, any treatment with potential psychotropic properties or interference with participants’ safety or data interpretation, pregnancy, or other scanning contraindications.

Ethical approval was granted by London-Bromley Research Ethics Committee (reference: 13/LO/1897). All participants provided informed written consent and received financial compensation.

### 1.2 JORT fMRI paradigm

**Figure 1.**
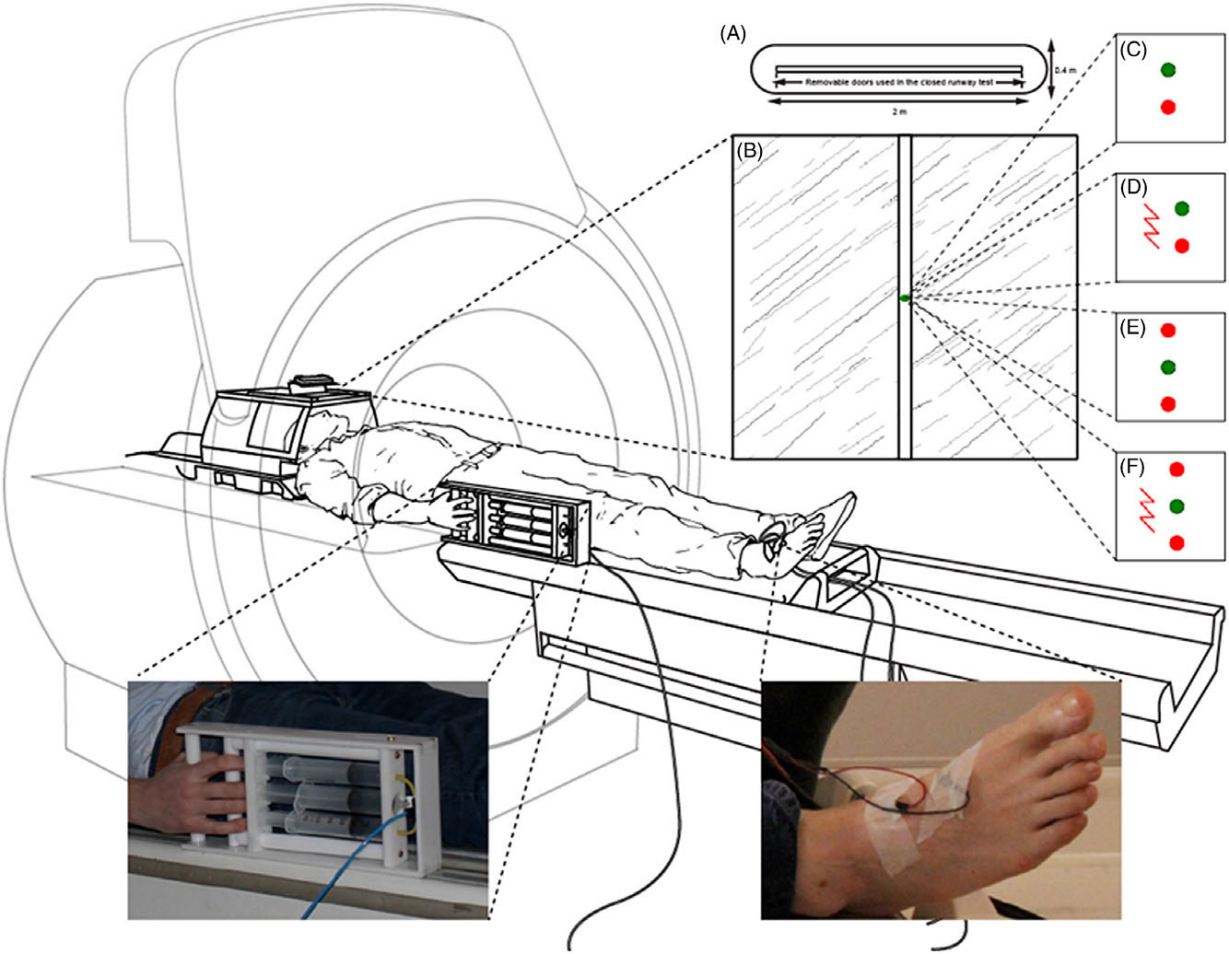
The fMRI Joystick Operated Runway Task (JORT). The JORT (B - F) is a human translation of the Mouse Defence Test Battery (A). As illustrated, participants squeezed a force-sensitive interface to control the speed of a green dot as it was pursued on the runway by red dot(s) (B). If the red dots collided with the green dot, on certain trials an electric shock was inflicted. The task comprised 12 trials of each type: C) Pursuit; D) Pursuit plus threat of electric shock; E) Goal-Conflict; and F) Goal-Conflict plus threat of electric shock.

The task is illustrated in Figure 1. In Pursuit trials (Figure 1C), the participant was instructed to squeeze a hand gripper to move a virtual agent cursor (a green dot) along a runway fast enough to remain ahead of the moving predatory red dot so as not to get caught. The gripper was force sensing (the greater the force applied to the handle, the faster the green dot would move), allowing the participant to control the dot’s speed. The force-sensing hand gripper was set to require a force of 7.5 kg to keep the green dot ahead of the red. This level was chosen from pilot testing of acceptability (effortful but not painful).

The goal-conflict trials comprised a second additional red dot which traveled above the green dot (Figure 1E). This required the participant to move the green dot fast enough to avoid the pursuing red dot but not too fast that it would collide with the leading red dot. Half of each set of trials had a lightening flash symbol displayed (Figure 1D and F) signaling that the participant would receive an electric shock to the right foot if the red dot(s) collided with the green dot. Before beginning the task, the participant calibrated the MRI-compatible electric shock machine to a level that they found aversive but not painful (capped at 80 Volts at 20 amperes).

Forty-eight trials in total (12 trials of each class) were presented in a pseudo-randomized order. Inter-trial intervals varied between 15–30 s to heighten unpredictability, during which time a fixation cross was presented. All participants underwent a practice session to familiarize them with the skill and force required to successfully complete the task.

#### 1.2.1 Behavioral measures of JORT performance

Behavioral measures of performance were calculated from the amount of pressure applied to the hand gripper and the resulting movement on the runway. Flight intensity (FI) in the JORT was calculated as average velocity (V) in the one-way active avoidance trials that contained no threat (Ta) of white noise subtracted from the average velocity in the one-way active avoidance trials with threat (Tp) of white noise. Thus, FI = VTa-VTp.

RAI in the JORT was calculated as standard deviation (SD) of the average velocity (V) in the two-way active avoidance trials that contained no threat (Ta) of white noise subtracted from the SD of the average velocity in the two-way active avoidance trials with threat (Tp) of white noise. Thus, RAI = V(SD).Ta-V(SD).Tp.

Additionally, the average velocity of movement was calculated for pursuit trials (both threat and non-threat) and the average oscillations (standard deviation of speed of movement) were calculated for goal-conflict trials (both threat and nonthreat conditions).

### 1.3 Psychological measures

*JORT dread rating* **:** after completing the JORT, participants rated how much dread they had experienced while the red dots were chasing them on a scale of 1 (no dread) to 10 (maximum dread).

*Neuroticism:* via Eysenck’s Personality Questionnaire – Revised Version (EPQ-R) (Eysenck, Eysenck, & Barrett, 1985). Higher scores indicate greater neuroticism.

*Trait anxiety:* via Spielberger’s State Trait Anxiety Inventory (STAI) (Spielberger, Gorsuch, & Lushene, 1970). Higher scores indicate greater trait anxiety.

*Fear:* via the fear of tissue damage subscale of the Fear Schedule Survey (FSS) (Wolpe & Lang, 1964). This subscale has been found to be related to FI and represents a relatively pure measure of fear (Perkins et al., 2011, 2013).

### 1.4 Image analysis

#### 1.4.1 fMRI acquisition

Structural and functional images were acquired on a 3-Tesla GE MR750 scanner with a 12-channel radiofrequency head coil. The structural sequence comprised a high-resolution sagittal Magnetisation Prepared Rapid Acquisition GRE 3D Inversion Recovery (MP-RAGE) anatomical reference image: inversion time = 400 ms (ms); echo time (TE) = 3.016 ms; repetition time (TR) = 7.312 s; flip angle 11°; slice thickness = 1.2 mm (196 contiguous slices). These T1-weighted gradient echo structural images were normalized and segmented into grey matter, white matter, and cerebrospinal fluid. The functional sequence comprised T2*-weighted gradient EPI sessions of 543 whole brain volume acquisitions: flip angle 75°; RT = 2000 ms; TE = 30 ms; FOV = 24 × 24 cm; slice-thickness = 3 mm; inter-slice gap = 0.3 mm (total of 41 slices); matrix size = 64 × 64 voxels with an isotropic 3 mm × 3 mm in-plane resolution for a total functional scan duration of approximately 18 minutes.

#### 1.4.2 fMRI pre-processing

Pre-processing was conducted using Statistical Parametric Mapping, Version 12 (SPM-12). Images were realigned to the first image of the run, slice timing was corrected, and functional images were co-registered to the T1 image. Segmentation and normalization were performed in SPM using the default approach and parameters, and deformation fields were then used to normalize the functional images to MNI space. Realignment parameters were inspected and subjects demonstrating translation over one voxel were excluded from further analysis. The first four volumes from each session were discarded to allow for magnetization equilibrium.

#### 1.4.3 First level analysis

**Figure 2.**
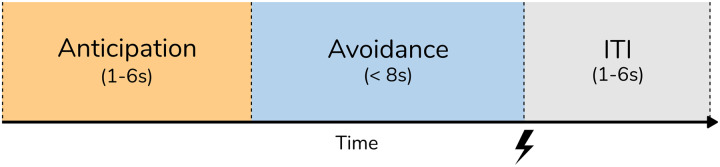
Illustration of trial timings. ITI, Inter-trial interval; S, seconds. The task was 18 min and 14 s in total.

First level analysis was performed using SPM-12 using a general linear model. Regressors for each trial type were included for Anticipation (the time preceding the start of the chase when the type of trial was cued), Active-Avoidance (time during the chase by the red dot(s)), and the End of the Chase (split into trials where the participant was caught and those where they escaped). See Figure 2 for an illustration of trial timings.

The following parametric modulators were included: 1) Cumulative Threat, the area under the curve of the participants’ distance from the closest chasing stimulus, representing how close the subject was to getting caught on average; 2) Peak Threat, the closest distance to either chasing stimulus during the trial; 3) Oscillation Amplitude (anxiety-related behavior), defined as the standard deviation of the participant’s movement.

Given the risk of head motion induced by the electric shocks in the task, ensuring non-neural motion-related artifacts in the data did not influence the results was a priority. In addition to including motion regressors in pre-processing steps, time points exhibiting high levels of motion were identified using the motion outliers tool included in the FMRIB software library (FSL, https://fsl.fmrib.ox.ac.uk/fsl/fslwiki/) based on Derivative of rms VARiance over voxels (DVARS; which is a measure of how much the intensity of a brain image changes from one volume to the next) and framewise displacement measures (head movement from one volume to the next) (Power, Barnes, Snyder, Schlaggar, & Petersen, 2012). Regressors for these time points were included in first-level modules to exclude them from model estimation. We also used CompCor (Behzadi, Restom, Liau, & Liu, 2007) to identify signals in the data likely representing signals of non-neural origin. Briefly, this involves extracting signal from white matter and cerebrospinal fluid voxels, based on segmented T1 images, before using principal component analysis (PCA) to reduce the dimensionality of this data and produce a chosen number of components (six in this case) representing non-neural signals. These component time series were then included in first-level models to reduce the impact of both motion and physiological arousal.

Occasionally participants failed to react to the trial or a technical issue with the force sensor on the gripper led to an absence of movement. These trials were excluded. Participants whose data were unusable for more than four trials of any condition were excluded from analyses. One participant’s data was removed due to excessive head movement and a further two were removed due to more than four trials with unusable data.

Smoothing was applied to the contrast images with an 8 mm full width at half maximum Gaussian kernel.

#### 1.4.4 Second-level analysis

Our second-level analyses were purposefully straightforward; all relevant task effects were modeled at the first level, providing maps that were taken forward to second-level analysis to be analyzed using *t*- and F-tests. This allowed us to simplify the second-level modeling process, avoiding the need for complex mixed factorial models that have additional statistical assumptions that must be met (e.g., sphericity). As described above, first-level analyses provided maps for each condition of interest in the task, in addition to the effect of parametric modulators. We then used one-sample F-tests on these first-level maps to identify main effects of the task. Main task effects were evaluated in the control and MDD groups together to allow identification of systems involved on the task overall. Group comparisons (MDD versus healthy controls) were performed using independent t-tests on these same maps. All analyses included total distance traveled derived from the realignment parameters, an index of subject-level head motion, as a covariate. Group comparisons also included age and gender as covariates to ensure that demographic differences between groups did not influence our results, even if differences in demographics between subjects did not reach significance.

The primary contrasts were the task conditions (anticipation and active avoidance), both compared to the baseline resting condition (fixation on a cross). The following neural main-effects were explored in both anticipation and active-avoidance task conditions: 1) pursuit versus goal-conflict conditions; and 2) threat versus no threat (safe) trials. Additionally, in the active avoidance phases of the task, we tested whether the activity was associated with (1) RAI; and (2) how close the red predator(s) were, giving a measure of brain activity in peak threat. Note that each of these effects was modeled at the first level, with group-level significance assessed using F-tests at the second level.

In addition to evaluating the main effects of the task and differences between groups, we also sought to identify associations between task-induced activity and individual difference measures. Regressions explored the relationship between individual differences in psychological variables and neural activity in the anticipation and avoidance phases. These regression analyses were conducted as whole-brain analyses in SPM. These included post-scan ratings of subjective dread, neuroticism, trait anxiety (STAI), and fear (FSS, tissue damage subscale). Activity during the anticipation phase was correlated with trait anxiety and dread rating. We also included regression models investigating how these measures related to the interaction between threat versus no threat trials during the anticipation phase to evaluate threat-specific effects. In the active-avoidance phase, relationships between self-report trait anxiety and neural activation in goal-conflict trials were explored. We also assessed relationships between self-report fear and activation on pursuit trials.

For the exploratory whole-brain analyses, results were thresholded with a voxelwise, cluster-defining threshold of *p* < .001 and a cluster-level threshold of *p* < .05, family-wise error (FWE) corrected (Nichols & Hayasaka, 2003). We report significant clusters in the results section. The bilateral hippocampal ROIs were generated using the WFU-Pickatlas toolbox using automatic anatomical labeling (AAL). The PAG ROI was defined as per Mobbs et al. (2007, 2009) with a 6 mm radius, *x* = 4, *y* = −30, *z* = −24 in MNI space. ROI analyses were performed by applying small volume correction to the whole-brain maps to identify clusters within these regions of interest, without the requirement to correct across the whole brain.

### 1.5 Behavioral analysis

Data were analysed using SPSS Version 24.0 (IBM Corp, 2016). Differences in group performance were assessed with independent-samples t-tests. Associations between JORT RAI, FI, velocity on pursuit trials, and oscillations on goal-conflict trials and other measures relevant to threat avoidance (the FSS-tissue damage subscale, STAI, neuroticism, and dread rating) were assessed with Pearson correlation coefficients.

## 2. Results

Eighteen individuals with MDD and 17 controls were included; see Table 1 for participant characteristics and behavioral JORT performance. There were no significant differences between groups on the JORT’s behavioral measures; however, participants with MDD reported experiencing significantly more dread, *t*(33) = 3.8, *p* < .001.

**Table 1.** Participant characteristics and JORT performance

	MDD Group (*n* = 18)	Control Group (*n* = 17)	Comparison (MDD versus controls)
Age (years)	30.4 (9.2)	32.4 (10.7)	*t* = −0.6
Gender (M/F)	7/11	7/10	χ^2^ = 0.9
Comorbidities (n)^a^	GAD (9), SAD (6), OCD (4), PTSD (2), PD (1)	–	–
Depression severity (HDRS)	19.1 (3.8)	0.7 (1.1)	*t* = 19.7**
Trait anxiety (STAI)	62.1 (7.8)	33.4 (6.1)	*t* = 12.0**
Dread Score (0–10)	5.4 (2.8)	1.9 (2.7)	*t* = 3.8**
Neuroticism (EPQ)	20.3 (3.5)	6.8 (5.1)	*t* = 9.2**
Fear (FSS-TD)	49.0 (29.0)	23.9 (18.0)	*t* = 3.1*
JORT FI	0.18 (0.73)	−0.07 (0.86)	*t* = 1.0
JORT RAI	−1.7 (0.42)	−0.14 (0.27)	*t* = −0.3
Average speed on pursuit trials	9.0 (3.3)	8.1 (2.8)	*t* = −0.6
Average oscillations on goal-conflict trials	3.1 (0.5)	3.2 (0.4)	*t* = 0.88

MDD, major depressive disorder; M, Male; F, Female; GAD, generalized anxiety disorder; SAD, social anxiety disorder; OCD, obsessive compulsive disorder; PTSD, post-traumatic stress disorder; PD, panic disorder; HDRS, 17-item Hamilton Depression Rating Scale; STAI, State Trait Anxiety Inventory; FSS-TD, Fear Schedule Survey tissue damage; JORT, Joystick Operated Runway Task; FI, Flight Intensity; RAI, Risk Assessment Intensity.

a Non-exclusive; 66.7% met criteria for ≥1 comorbid diagnosis.

Values are reported as mean (standard deviation) except where otherwise stated. Comparison was by independent samples t-tests or Pearson chi-square for categorical variables. * Significant to *p* < .05 ** Significant to *p* < .001.

### 2.1 Anticipation phase

#### 2.1.1 Neural activity overall

The anticipation phase was associated with significantly elevated activation in two clusters including the right putamen/right anterior insula and left superior occipital gyrus/left cuneus and significantly reduced activation compared to baseline in right occipital, superior, and temporal gyri, all *p* < .001 (Table 2).

**Table 2.** Brain activation during the anticipation phase of the JORT

Brain region	MNI Peak co-ordinates	*p*	Cluster size (voxels)	F
*Anticipation (main effect) > Baseline*				
Right putamen (bordering right anterior insula)	24, 10, 6	<.001	4058	133.0
Left superior occipital gyrus / left cuneus	−18, −76, 34	<.001	9835	68.5
*Baseline > Anticipation (main effect)*				
Right occipital fusiform gyrus	22, −88, −10	<.001	7270	66.2
Right superior/middle temporal gyrus	46, −16, −12	<.001	1284	36.4
*Threat (main effect) > no threat*				
Left ACC / superior frontal gyrus	−16, −6, 51	<.001	881	34.8
Right Caudate	20, 26, 2	<.001	2196	32.2
Right superior frontal gyrus / right pre-supplementary motor area	16, −4, 54	.022	378	30.3

ACC, anterior cingulate cortex; JORT, Joystick Operated Runway Task; MNI, Montreal Neurological Institute.

*N* = 35 (18 participants with MDD and 17 controls). Significance was FWE cluster corrected. Peak coordinates are reported in MNI space.

#### 2.1.2 Goal-conflict versus pursuit trials

There were no significant main effects of neural activation in the anticipation phase correlating with condition type (i.e., goal-conflict versus pursuit trials).

#### 2.1.3 Threat versus no threat trials

There was a main effect of threat on neural activity: trials signaling an electric shock were associated with elevated activity in the left superior frontal gyrus/dorsal anterior cingulate cortex (dACC) (*p* < .001), right caudate (*p* < .001), right superior frontal gyrus, and supplementary motor area (*p* = .022).

#### 2.1.4 MDD versus controls

There were no significant results for group comparisons on any contrasts during the anticipation phase.

#### 2.1.5 Correlations between neural activity, behavioral, and psychological measures

**Figure 3.**
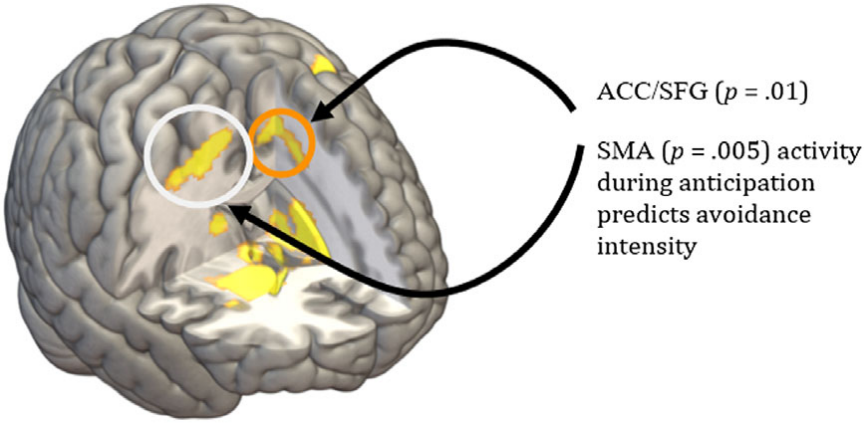
Brain activation during anticipation of threat (main effect of threat during anticipation, *p* < .05 FWE). ACC, anterior cingulate cortex; SFG, superior frontal gyrus; SMA, supplementary motor area.

**Figure 4.**
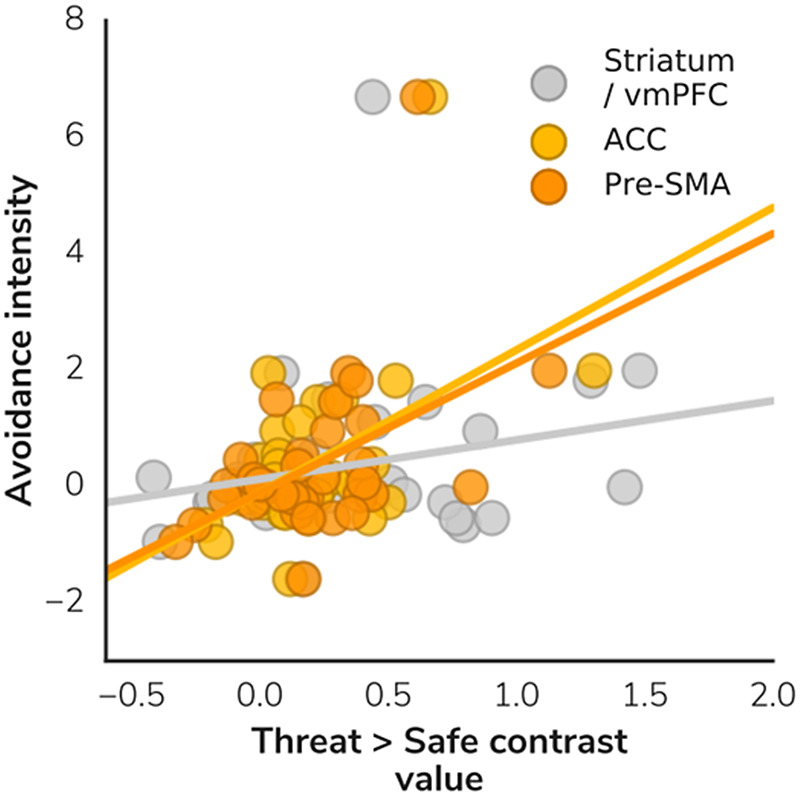
Correlation between avoidance intensity (speed of movement) and threat during the anticipation phase of the JORT. vmPFC, ventromedial prefrontal cortex; ACC, anterior cingulate cortex; pre-SMA, pre-supplementary motor area.

There were no correlations between self-report measures (neuroticism, trait anxiety, or subjective dread rating) and neural activity during anticipation. However, activity in the pre-supplementary motor area and dACC/superior frontal gyrus was positively correlated with avoidance intensity (i.e., speed of movement) and threat level in the anticipation phase (*p* = .010 and .005, respectively); see Figures 3 and 4.

### 2.2 Active-avoidance phases

#### 2.2.1 Neural activity overall

The main effect of both active-avoidance conditions (pursuit and goal-conflict trials combined) compared to baseline fixation showed elevated activation in the right precuneus/superior parietal lobule and right middle frontal gyrus (Table 3).

**Table 3.** Brain activation during active threat avoidance (pursuit and goal-conflict)

Brain region	MNI Peak co-ordinates	*p*	Cluster size (voxels)	F
*Avoidance (main effect) > baseline*				
Right precuneus / superior parietal lobule	14, −52, 58	<.001	62,177	339.1
Right middle frontal gyrus	34, 40, 32	<.001	1464	82.3
*Baseline > avoidance (main effect)*				
Left occipital pole, left calcarine cortex, left occipital fusiform gyrus	−16, −94, −6	<.001	797	60.4
Left precuneus / posterior cingulate gyrus	−8, −54, 20	<.001	3043	102.6
Left angular gyrus	−44, −64, 30	<.001	1485	90.5
Left anterior orbitofrontal cortex	−26, 32, −16	<.001	4268	54.3
Right lingual gyrus and right occipital fusiform gyrus	20, −88, −8	.002	550	48.1
Left superior / middle frontal gyrus	−20, 24, 44	<.001	810	36.1
*Pursuit > goal-conflict (main effect)*				
Left cerebellum	0, −66, −34	<.001	30,141	67.69
Right temporal and superior cortex	50, −32, 20	<.001	3290	51.40
Right anterior insula	40, 18, −10	.001	712	36.02
*Goal-conflict > pursuit (main effect)*				
Left anterior orbitofrontal cortex	−22, 38, −10	<.001	1531	39.43
*Threat (main effect) > no threat*				
Right posterior insula/ right hippocampus	40, −22, −4	.041	276	20.47
*No threat > threat (main effect)*				
Left middle/superior temporal gyrus	−28, 56, 12	.004	562	37.07
Left caudate	−16, 24, 2	.041	269	35.62
Left frontal superior gyrus	−12, 38, 38	.005	489	35.17
Right postcentral gyrus	42, −30, 48	.003	661	29.87
*Avoidance (main effect) > baseline: Positive correlations with oscillation in movement made*				
Right precuneus, right superior parietal lobule	14, −52, 58	<.001	68,267	209.09
Right mid frontal gyrus	38, 44, 22	<.001	1413	49.88
Left middle frontal cortex	−40, 38, 32	.007	375	29.82
*Avoidance (main effect) > baseline: Negative correlations with oscillation in movement made*				
Left & right precuneus and posterior cingulate	−8, −52, 18	<.001	2060	107.12
Left angular gyrus, left mid temporal cortex	−44, −62, 28	<.001	993	71.01
Left occipital cortex	−16, −96, −4	.002	537	46.34
Right lingual gyrus, right occipital fusiform gyrus, right calcarine cortex	18, −88, −8	.011	329	45.02
Left anterior cingulate, left medial frontal cortex	−6, 44, −8	<.001	1255	34.40
Left temporal cortex	−56, −44, −14	.005	424	31.17
Left orbitofrontal cortex (anterior / medial and posterior orbital gyrus)	−24, 34, −12	.013	306	29.81

MNI, Montreal Neurological Institute.

*N* = 35 (18 participants with MDD and 17 controls). Significance was FWE cluster corrected. Peak coordinates are reported in MNI space.

#### 2.2.2 Goal-conflict versus pursuit trials

A comparison between the active avoidance conditions (goal-conflict versus pursuit trials) showed significantly elevated activation in the left cerebellum (*p* < .001), right temporal and superior cortex (*p* < .001), and right anterior insula (*p* = .001) in pursuit trials compared to goal-conflict trials and elevated left anterior orbitofrontal cortex activation in goal-conflict compared to pursuit trials (*p* < .001).

#### 2.2.3 Threat versus no threat trials

There was a main effect of threat, with threat trials associated with significantly elevated activation in a cluster including the right insula and hippocampus (*p* = .041). No-threat trials were associated with elevated activation in prefrontal regions (all *p* < .01) and the caudate (*p* = .041).

#### 2.2.4 MDD versus controls

There were no significant results for group comparisons (MDD versus controls) on any of the contrasts during the active avoidance phases. There were no significant differences between the groups in the number of times they were caught by the chasing predators (*t*(33) = 1.04, *p* = .30), suggesting that ability did not confound results.

#### 2.2.5 Correlations between neural activity, behavioral, and psychological measures

**Figure 5.**
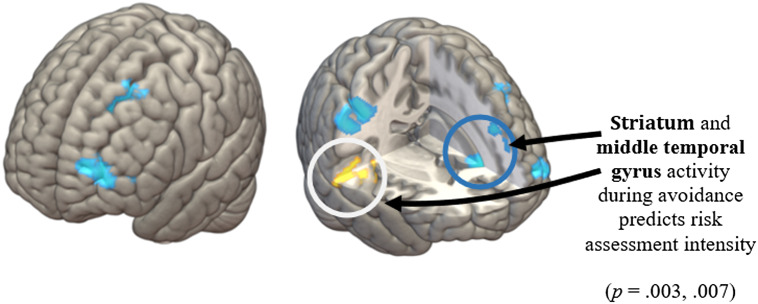
Brain activation during active avoidance (main effect of threat during avoidance, *p* < .05 FWE).

**Figure 6.**
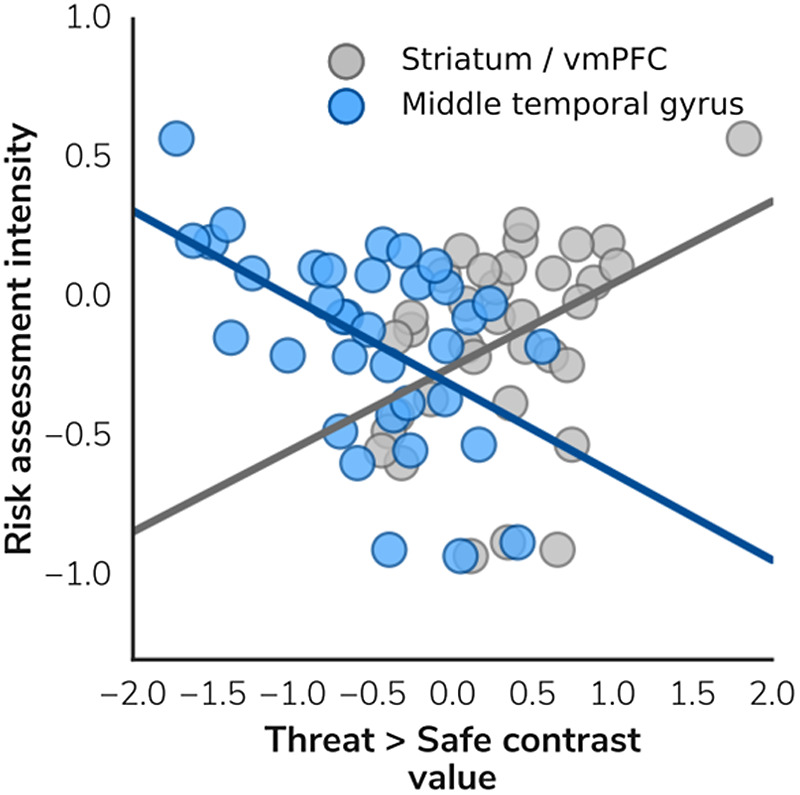
Correlation between Risk Assessment Intensity and neural activation in the active avoidance phase of the JORT. vmPFC, ventromedial prefrontal cortex.

There was no significant main effect of peak threat (i.e. no correlation between activation with proximity of the red dot predator(s) to the green dot agent); however, RAI was significantly associated with striatum and middle temporal gyrus activity (*p* = .003 and .007, respectively); see Figures 5 and 6. There were no further significant correlations with psychological variables or JORT behavioral measures with brain activation for the main contrasts.

## 3. Discussion

This study presents a psychiatric validation of the JORT, a measure of threat avoidance that allows within-task, within-subject comparison of fear and anxiety (Perkins et al., 2009, 2011, 2019). Neural main effects were found for both the anticipation and active avoidance phases of the task, as well as for pursuit versus goal-conflict trials. No differences were found between participants with MDD and healthy controls on the neural or behavioral level, though participants with MDD did report higher levels of dread while being chased on the task. Further, brain activity and JORT behavioral measures did not correlate with self-report measures of threat sensitivity, in contrast with our hypotheses.

Results suggest that the JORT was effective in identifying neural systems involved in anticipation and active avoidance of threat. Anticipation was associated with significant activation in the ACC/superior frontal gyrus, insula, and striatum, while active avoidance was associated with activity in prefrontal regions, the dACC and insula, in line with previous research (Mobbs et al., 2007, 2009; Rzepa et al., 2017). Our findings align with previous research suggesting that the ACC, supplementary motor area, and striatum are activated with threat anticipation (Mobbs et al., 2007; Rzepa et al., 2017). Mobbs et al. (2007) found that dACC was related to imminent, as opposed to distal, threats supporting this observed relationship with avoidance intensity. Our additional finding of supplementary motor activation during anticipation fits with other research involving threats of electric shocks (Maresh, Beckes, & Coan, 2013).

Our results also demonstrate differential brain activation in response to threat versus no threat trials in the JORT. The elevated PFC activation found in low threat trials may signify that participants were more able to engage in higher order cognitive appraisal of threat avoidance when there was no risk of receiving an electric shock, whereas in the presence of threat (shock), elevated insula, and hippocampal activation may signify increased emotional reactivity, as would be expected in this condition (Mobbs & Kim, 2015). These results differ from the pilot fMRI JORT study, where no main effects of threat were found (Perkins et al., 2019). This difference could be due to our inclusion of participants with MDD and the resulting intra-sample variability in sensitivity to threat. Indeed, the original pilot sample scored on average one standard deviation below normal on neuroticism.

Although results suggested differentiation in the neural systems that govern goal-conflict (anxiety-related) and simple threat avoidance (pursuit, fear-related), these were not in the hypothesized regions. Based on previous research (Mobbs et al., 2007, 2009), it was hypothesized that pursuit trials would activate midbrain regions, e.g., the PAG. However, this finding was not replicated either at a whole brain or ROI level, instead finding that pursuit trials were associated with elevated activation in the left cerebellum, left orbitofrontal cortex, and right anterior insula. Additionally, the hippocampal ROI analysis did not show an association with goal-conflict conditions unlike previous findings (Abraham et al., 2013; Bach et al., 2014; O’Neil et al., 2015). Further, no correlations with behavioral measures of JORT performance or psychological variables were found on main effects in either the anticipation or active avoidance phases. This limits our ability to draw conclusions regarding the defensive direction hypothesis, whereby anxiety and fear activate distinct brain regions and are associated with trait fear and anxiety measures. It may be that pathological responses separating anxiety and fear are less clearly defined in humans compared to the animal models upon which the theory was developed.

A strength of the JORT is that unlike most threat-sensitivity paradigms, the task allows for the effect of threat imminence to be explored. Most tasks, for example, Pavlovian conditioning paradigms, do not vary in proximity (Büchel & Dolan, 2000). The work of Mobbs et al., (2007, 2009) has been instrumental in showing the effects of threat imminence on human brain activity during pursuit trials – finding that the midbrain takes over from prefrontal activation when threats are near. The JORT has the advantage of also exploring goal-conflict, within-task, and within-subjects. In contrast to expectations and previous findings in healthy controls, no association between threat imminence and brain activity, specifically the PAG, was found. It may be that the relatively small sample size lacked the power to find such effects. Additionally, the tasks used by Mobbs et al. (2009) involved the loss of a potential reward if caught, a feature not included in the JORT.

No significant differences in neural activation on the task between participants with MDD and controls were found. Comorbid anxiety disorders, or overlapping symptoms between MDD and anxiety, may account for the relationship between threat sensitivity and depression. Indeed, a meta-analysis showed that trait anxiety and fear of anxiety-related situations and threats were mostly associated with agoraphobia, general anxiety disorder, panic disorder, and post-traumatic stress disorder compared to MDD (Naragon-Gainey, 2010). However, previous research has shown that the relationship between threat avoidance and depression remains after controlling for comorbid anxiety (Johnson, Turner, & Iwata, 2003). Another possibility is that our relatively small sample size limited our ability to find significant differences between groups.

The JORT is an active, signaled avoidance task. Future research should investigate the neural basis of passive avoidance (tasks which involve withholding of behaviors to avoid aversive events) in MDD, as self-report data have shown elevated levels of passive avoidance in this population (Pinto-Meza et al., 2006). Additionally, studies using tryptophan depletion have demonstrated that serotonin depletion in healthy control participants results in reduced responses to punishments in passive avoidance tasks (Finger et al., 2007).

### 3.1 Limitations

As with all threat-avoidance paradigms, the JORT cannot fully represent naturalistic threats. There is a higher-order nature of the threat of electric shocks in relation to the pursuit/goal-conflict scenarios presented on the screen, i.e., the threat avoidance is symbolic rather than ecologically natural. Although the JORT is not analogous to real-world threatening events, the results show alignment with other threat avoidance and anticipation studies in humans and also the animal literature where highly replicable circuits are found, increasing our confidence that threat avoidance is measured successfully by the task (Gray & McNaughton, 2000; McNaughton & Corr, 2004; Mobbs & Kim, 2015). The physical effort required on the JORT, unlike many tasks of threat avoidance in humans, adds to the task’s ecological validity.

Other fMRI paradigms designed to measure goal conflict have involved a conflict between a reward (e.g., a monetary incentive) versus punishments (e.g., threat of losing rewards) (Bach et al., 2014; Gonen et al., 2016; Mobbs et al., 2013). The JORT does not involve a reward to successfully negotiate the threat. In depression, reduced sensitivity to rewards and an enhanced focus on punishments are reported (Eshel & Roiser, 2010), and the avoidance observed in the condition may be related to decreased reward sensitivity more than active avoidance of perceived threat. A task additionally involving rewards may therefore capture more of the underlying cognitive and neural differences in MDD than a task which simply involves avoiding punishments. Finally, although multiple comparisons were corrected for within analyses, a relatively large number of regressions were performed without correction across analyses. Given that this is the first study to use the JORT in a clinical population, we emphasize that future studies with larger sample sizes are required to further validate the JORT as a measure of threat avoidance.

## 4. Conclusions

The current study administered the fMRI version of the JORT in participants with MDD. Results suggest that the JORT was effective in identifying neural regions involved in avoidance and anticipation of aversive stimuli, with activation being linked to threat level. However, no significant differences between participants with MDD and healthy controls were found on a neural or behavioral level. The task should additionally be explored in patients with anxiety disorders, where stronger associations have been found with threat-avoidance behaviors (Naragon-Gainey, 2010). Tasks measuring deficits in reward processing via passive avoidance tasks may be more relevant to MDD psychopathology (Ferster, 1973; Ottenbreit & Dobson, 2004).
